# Adulterated Medicines

**Published:** 1849-11

**Authors:** 


### ADULTERATED MEDICINES.

At the last meeting of the American Medical Association a committee of two members from each State and Territory was appointed, whose duty it was made "to note all the facts that come to their knowledge with regard to the adulteration and sophistication of Drugs, Medicines, Chemicals, &c., and to report them through the chairman at the next annual meeting." Prof. Huston, of Philadelphia is chairman of the committee. The subject is one which the Association deemed of great importance, and which we hope will receive at the hands of the committee a thorough investigation. We have to ask of our readers to communicate to us any facts in relation to it which may come to their knowledge. If we can excite the attention of practitioners generally in reference to the matter, we may hope to ascertain whether the medi-icines used by them are of the proper character.

